# A detailed characterization of drug resistance during darunavir/ritonavir monotherapy highlights a high barrier to the emergence of resistance mutations in protease but identifies alternative pathways of resistance

**DOI:** 10.1093/jac/dkad386

**Published:** 2023-12-28

**Authors:** Adam Abdullahi, Ana Garcia Diaz, Olga Mafotsing Fopoussi, Apostolos Beloukas, Victoire Fokom Defo, Charles Kouanfack, Judith Torimiro, Anna Maria Geretti

**Affiliations:** Takemi Program in International Health, Harvard T.H. Chan School of Public Health, Boston, MA, USA; Cambridge Institute of Therapeutic Immunology & Infectious Disease, Cambridge, UK; Institute of Human Virology Nigeria, Abuja, Nigeria; Department of Virology, Royal Free London NHS Foundation Trust, London, UK; Biomedical Sciences Department, University of West Attica, Athens, Greece; Chantal Biya International Reference Centre for Research on HIV/AIDS Prevention & Management (CIRCB), Yaoundé, Cameroon; Biomedical Sciences Department, University of West Attica, Athens, Greece; National AIDS Reference Centre of Southern Greece, School of Public Health, University of West Attica, Athens, Greece; Chantal Biya International Reference Centre for Research on HIV/AIDS Prevention & Management (CIRCB), Yaoundé, Cameroon; Department of HIV Medicine, Hôpital Central de Yaoundé, Ministry of Public Health, Yaoundé, Cameroon; Department of HIV Medicine, Hôpital Central de Yaoundé, Ministry of Public Health, Yaoundé, Cameroon; Chantal Biya International Reference Centre for Research on HIV/AIDS Prevention & Management (CIRCB), Yaoundé, Cameroon; Department of Infectious Diseases, Fondazione PTV, University of Rome Tor Vergata, Rome, Italy; Department of Infection, North Middlesex University Hospital, London, UK; School of Immunity and Microbial Sciences, King’s College London, London, UK

## Abstract

**Background:**

Maintenance monotherapy with ritonavir-boosted darunavir has yielded variable outcomes and is not recommended. Trial samples offer valuable opportunities for detailed studies. We analysed samples from a 48 week trial in Cameroon to obtain a detailed characterization of drug resistance.

**Methods:**

Following failure of NNRTI-based therapy and virological suppression on PI-based therapy, participants were randomized to ritonavir-boosted darunavir (*n* = 81) or tenofovir disoproxil fumarate/lamivudine +ritonavir-boosted lopinavir (*n* = 39). At study entry, PBMC-derived HIV-1 DNA underwent bulk Protease and Reverse Transcriptase (RT) sequencing. At virological rebound (confirmed or last available HIV-1 RNA ≥ 60 copies/mL), plasma HIV-1 RNA underwent ultradeep Protease and RT sequencing and bulk Gag-Protease sequencing. The site-directed mutant T375A (p2/p7) was characterized phenotypically using a single-cycle assay.

**Results:**

NRTI and NNRTI resistance-associated mutations (RAMs) were detected in 52/90 (57.8%) and 53/90 (58.9%) HIV-1 DNA samples, respectively. Prevalence in rebound HIV-1 RNA (ritonavir-boosted darunavir, *n* = 21; ritonavir-boosted lopinavir, *n* = 2) was 9/23 (39.1%) and 10/23 (43.5%), respectively, with most RAMs detected at frequencies ≥15%. The resistance patterns of paired HIV-1 DNA and RNA sequences were partially consistent. No darunavir RAMs were found. Among eight participants experiencing virological rebound on ritonavir-boosted darunavir (*n* = 12 samples), all had Gag mutations associated with PI exposure, including T375N, T375A (p2/p7), K436R (p7/p1) and substitutions in p17, p24, p2 and p6. T375A conferred 10-fold darunavir resistance and increased replication capacity.

**Conclusions:**

The study highlights the high resistance barrier of ritonavir-boosted darunavir while identifying alternative pathways of resistance through Gag substitutions. During virological suppression, resistance patterns in HIV-1 DNA reflect treatment history, but due to technical and biological considerations, cautious interpretation is warranted.

## Introduction

HIV drug-resistant variants acquired at the time of infection or enriched under selective drug pressure can integrate into the DNA of memory CD4 T-cells as provirus and become part of the HIV DNA archive within the host cell.^1,2^ Archived resistant variants can re-emerge if virus production resumes, thus potentially retaining life-long clinical significance. While the available evidence is not completely consistent, studies have demonstrated that detecting drug resistance-associated mutations (RAMs) in the HIV-1 DNA of virologically suppressed patients predicts virological outcomes when switching to a different treatment regimen.^2,3^ Interestingly, some studies have produced surprising findings. We previously reported that detecting archived RAMs during suppressive ART with two NRTIs plus a ritonavir-boosted PI was associated with a reduced likelihood of virological rebound after switching to ritonavir-boosted darunavir monotherapy.^4^

Maintenance monotherapy with ritonavir-boosted darunavir has been studied in Western Europe among virologically suppressed patients without previous treatment failure.^5^ A multicentre study conducted in Burkina Faso, Cameroon and Senegal investigated ritonavir-boosted darunavir monotherapy among 50 patients who had achieved virological suppression on ritonavir-boosted PI-based triple ART after failure of two NRTIs plus an NNRTI.^6^ These studies uniformly reported an increased risk of viraemia when switching to ritonavir-boosted darunavir monotherapy, and also consistently demonstrated a low risk of treatment-emergent darunavir RAMs.

We conducted a randomized clinical trial in Cameroon comparing ritonavir-boosted darunavir maintenance monotherapy with standard of care (SOC) triple ART with two NRTIs plus ritonavir-boosted lopinavir in adults living with HIV. Similar to the study from Burkina Faso, Cameroon and Senegal,^6^ participants were virologically suppressed on ritonavir-boosted PI-based triple ART having experienced virological failure of first-line NNRTI-based therapy. We previously reported the virological outcomes of the ritonavir-boosted darunavir arm in relation to the detection of RAMs in cellular HIV-1 DNA at study entry.^4^ The aim of this analysis was to take advantage of unique trial samples and conduct a detailed characterization of the virological outcomes of the entire trial population. We performed a sensitive assessment of treatment-emergent resistance using ultradeep sequencing (UDS), including the evaluation of Gag mutations, and compared the resistance patterns detected in cellular HIV-1 DNA at study entry, when participants showed virological suppression, with those observed in plasma HIV-1 RNA during subsequent virological rebound.

## Methods

### Study population

Monotherapy in Africa, New Evaluations of Treatment (MANET) was a randomized, open-label trial based at Hôpital Central Yaoundé in Cameroon (NCT02155101). Between August 2014 and July 2015, 120 adults receiving virologically suppressive ART with two NRTIs plus a ritonavir-boosted PI (typically ritonavir-boosted lopinavir) were randomized to either ritonavir-boosted darunavir monotherapy (800/100 mg once daily) for 48 weeks (*n* = 81) or SOC with tenofovir disoproxil fumarate co-formulated with lamivudine plus ritonavir-boosted lopinavir for 24 weeks (*n* = 39). Eligibility criteria comprised having received two NRTIs plus a ritonavir-boosted PI for ≥12 weeks, CD4 count > 100 cells/mm^3^, plasma HIV-1 RNA < 60 copies/mL in two screening measurements taken 4–12 weeks apart, negative hepatitis B surface antigen (HBsAg) and absence of significant disease or laboratory abnormalities. Pregnancy or planning to become pregnant were exclusion criteria. Following randomization, participants of both arms attended scheduled study visits at Weeks 4, 12 and 24; the ritonavir-boosted darunavir arm also attended scheduled study visits at Weeks 36 and 48. The study was approved by the University of Liverpool Ethics Committee (RETH000605) and the Cameroon National Ethics Committee (2013/07/347) and overseen by an independent trial safety board.

### Statistical analysis

The characteristics of the population at study entry, summarized as categorical and continuous variables, were compared by chi-squared test, Fisher’s exact test or Wilcoxon Mann–Whitney test, as appropriate. The trial primary endpoint was the proportion of participants with plasma HIV-1 RNA < 400 copies/mL at Week 24 (FDA snapshot). Secondary virological endpoints measured through to Week 24 in both arms and through to Week 48 in the ritonavir-boosted darunavir arm comprised proportions with plasma HIV-1 RNA < 60 copies/mL and emergence of RAMs in participants with virological rebound, defined as confirmed (or last available) HIV-1 RNA ≥ 60 copies/mL. Virological failure was defined as confirmed (or last available) HIV-1 RNA ≥ 400 copies/mL.

### Laboratory tests

At the Centre Pasteur of Cameroon in Yaoundé, safety parameters and CD4 cell counts were measured using freshly collected samples. Plasma was separated from whole venous blood in EDTA within 2 h of collection and stored at −80°C. HIV-1 RNA was quantified with the Biocentric assay (Bandol, France; lower limit of quantification 60 copies/mL). At the Chantal Biya International Reference Centre for Research on HIV/AIDS Prevention & Management in Yaoundé, PBMCs were isolated by Ficoll-Hypaque gradient centrifugation and stored at −80°C. Sanger sequencing of Protease (amino acids, aa 1–99) and Reverse Transcriptase (RT, aa 1–335) was performed as described.^7^ Sample aliquots were shipped frozen to the UK for HIV-1 RNA sequencing (see below) and for the quantification of total HIV-1 DNA in PBMCs by real-time PCR as described.^8^

### HIV-1 RNA sequencing

Plasma samples from participants experiencing virological rebound underwent UDS as previously described.^9,10^ Briefly, samples with HIV-1 RNA < 10 000 copies/mL were enriched by ultra-centrifugation at 35 000 rpm for 20 min at 4°C; following extraction with the QIAamp viral RNA kit (QIAGEN, UK), a 1300 bp amplicon was generated covering Protease (aa 1–99) and RT (aa 1–335), purified with Agencourt AMPure XP magnetic beads (Beckman Coulter, UK) and quantified by the Qubit dsDNA High Sensitivity Assay Kit on the Qubit 3.0 fluorometer (Invitrogen, UK). A DNA library was prepared with the Nextera XT DNA Sample Prep Kit (Illumina, USA), followed by sequencing with the Illumina MiSeq Reagent Kit v2. After checking for quality, reads were analysed applying a frequency threshold of 1% and described as low frequency variants (representing 1%–14% of the variants in a sample) and high-frequency variants (≥15%). Using plasma samples from participants experiencing virological rebound on ritonavir-boosted darunavir, after extraction with the QIAamp Viral RNA kit (QIAGEN), viral RNA was reverse transcribed using the Superscript III One-Step RT PCR Kit with Platinum^®^ Taq High Fidelity enzyme followed by amplification using Platinum^®^ PCR SuperMix High Fidelity (Invitrogen). A 2200 bp amplicon spanning *gag* and *protease* was generated by nested PCR as detailed in Table S1 (available as Supplementary data at *JAC* Online), followed by Sanger sequencing.

### Sequence analysis

Major RAMs in RT and Protease were defined according to the Stanford HIV Drug Resistance Database v9.4.1.^11^ Darunavir RAMs comprised V11I, V32I, L33F, I47V, I50V, I54L/M, T74P, L76V, I84V and L89V. Sequences were analysed for the presence of in-frame stop codons to indicate defective proviruses and screened for evidence of APOBEC3G (A3G) hypermutation using the Los Alamos Hypermut 2.0 tool.^12^ Phylogenetic analysis was used to investigate linkage between *pol* sequences as described.^13^ Briefly, for each FASTQ and FASTA *pol* sequence generated, 10 reference sequences were downloaded from GenBank,^14^ duplicate sequences were manually removed, and maximum-likelihood phylogenetic trees were constructed using RAxML version 8.^15^ Phylogenies were inferred with Figtree v1.4.4 with 1000 bootstrap replicates.^16^ Phylogenetic analysis was also used to confirm the HIV-1 subtypes. *Gag* sequences were aligned with the HIV-1 HXB2 reference sequence using MEGA v 6.06.^17^ Mutations were reported according to their association with PI exposure, which was pre-determined by comparing full-length Gag and Protease sequences from 200 PI-naive and 191 PI-experienced individuals.^18^ Mutations associated with PI exposure were those showing a significantly higher prevalence in the PI-experienced group by Fisher’s exact test with Bonferroni correction, using a *P* value threshold of <0.001 for mutations occurring at cleavage sites (p17/p24, p2/p7, p7/p1, p1/p6, p24/p2) and <0.0001 for mutations occurring in other regions of Gag. They included 14 cleavage site mutations [2 in p17/p24 (V128I, Y132F), 4 in p2/p7 (S373T, A374S, T375A, T375N), 3 in p7/p1 (A431V, K436R, I437V) and 5 in p1/p6 (L449F, S451T, S451R, R452S, P453T)], and 19 mutations in other regions of Gag [10 in p17 (L61I, I94V, K103R, K113Q, K114R, D121G, D121A, T122E, N126S, Q127K), 5 in p24 (T186M, T190I, A210S, E211D, S310T), 3 in p6 (F463L, T469I, P478Q) and 1 in p2 (T371Q)].

### Phenotypic characterization of the gag cleavage site mutation T375A

T375A was inserted by site-directed mutagenesis into the WT vector P8.9NSX, which contains the *protease* and *RT* sequences of the NL4-3 strain of HIV-1,^19^ using the QuikChange Multi Site-Directed Mutagenesis Kit (Stratagene, UK). The amplified DNA was enriched by digestion of the parental DNA with DpnI and XL1-blue and supercompetent cells were transformed with the digested DNA. The plasmidic DNA was isolated using QIAprep Spin Miniprep Kit (QIAGEN) and screened for the presence of T375A by Sanger sequencing. Susceptibility to darunavir, atazanavir, lopinavir, indinavir and saquinavir, and replication capacity were determined by a single-cycle assay in HEK293T cells as described.^19^ Virus replication in the presence of drug was determined by luciferase quantification 48 h post-infection relative to no-drug controls. The mean 50% inhibitory concentration (IC_50_) from three separate experiments was calculated and results expressed as fold-changes (FC) in IC_50_ compared with WT P8.9NSX. Viral replicative capacity was determined by luciferase quantification in the absence of drug, calculating the mean luciferase activity from ≥4 values within the linear range, and expressed as percentage relative to WT P8.9NSX.

## Results

### Study population

At study entry, the 120 participants had received ART for a median of 7.5 years, including prior first-line NNRTI-based ART for a median of 3.0 years and current second-line ritonavir-boosted PI-based ART for a median of 3.1 years (Table 1). Most (106/120; 88.3%) were receiving once-daily tenofovir disoproxil fumarate/lamivudine plus twice-daily ritonavir-boosted lopinavir. The characteristics of the two arms were similar overall, although the ritonavir-boosted darunavir arm showed higher levels of total HIV-1 DNA and higher prevalence of RAMs in HIV-1 DNA (Table 1). As per the eligibility criteria, prior to randomization all participants had shown a suppressed viral load (< 60 copies/mL) in two screening measurements, which were separated by a median of 7 weeks (range 6–12). No prior viral load measurements were available in the medical records.

**Table 1.** Characteristics of the population at study entry (*n* = 120)

Characteristic		Total	DRV/r arm	SOC arm	*P* value
Total number (%)		120 (100)	81 (100)	39 (100)	—
Female, *n* (%)		91 (75.8)	61 (75.3)	30 (76.9)	0.371
Age (years), median (IQR)		44 (38, 52)	45 (38–51)	45 (37–52.2)	0.457
BMI (kg/m^2^), median (IQR)		25.5 (22.1–29.1)	25.4 (21.8–28.4)	26.0 (22.5–29.2)	0.371
Haemoglobin (g/dL), median (IQR)		12.3 (11.6–13.2)	12.3 (11.6–13.2)	12.4 (11.6–13.5)	0.834
Estimated GFR ≥ 90 mL/min, *n* (%)		102 (85.0)	67 (82.7)	35 (89.7)	—
Estimated GFR = 60–89 mL/min, *n* (%)		18 (15)	14 (17.3)	4 (10.3)	0.417
Time since HIV diagnosis (years), median (IQR)		8.5 (5.8–10.4)	8.8 (5.9–11.1)	8.0 (5.5–9.8)	0.166
CD4 count (cells/mm^3^), median (IQR)		467 (341–618)	466 (341–615)	536 (394–687)	0.077
Nadir CD4 count, cells/mm^3^, median (IQR)		92 (37–172)	90 (37–167)	128 (29–194)	0.427
HIV-1 DNA, (log_10_ copies/10^6^ PBMCs), median (IQR)		2.9 (2.4–3.2)	2.9 (2.5–3.3)	2.7 (2.2–2.9)	0.021
Duration of exposure (years), median (IQR)^a^	Any ART	7.5 (5.3–9.4)	7.6 (5.3–9.8)	6.9 (4.9–9.2)	0.348
	TDF	2.9 (1.5–4.7)	2.9 (1.3–4.6)	1.9 (3.3–5.3)	0.274
	ZDV	2.9 (1.5–5.4)	2.4 (1.5–4.0)	3.0 (1.4–5.8)	0.135
	d4T	2.6 (1.3–4.4)	2.6 (1.3–4.0)	3.2 (1.3–4.4)	0.405
	NNRTI	3.0 (1.4–5.5)	3.1 (1.6–5.5)	3.0 (1.7–5.1)	0.411
	PI/r	3.1 (1.3–5.3)	3.2 (1.3–5.8)	3.1 (1.5–4.9)	0.951
ART regimen at study entry, *n* (%)	TDF/3TC + LPV/r	106 (88.3)	69 (85.2)	37 (94.8)	0.143
	ABC + ddI + LPV/r	6 (5.0)	5 (6.2)	1 (2.6)	—
	ZDV/3TC + LPV/r	5 (4.2)	4 (4.9)	1 (2.6)	—
	TDF/3TC + ATV/r	2 (1.7)	2 (2.5)	0 (0)	—
	TDF + ABC + LPV/r	1 (1.0)	1 (1.2)	0 (0)	—
≥1 RAM in HIV-1 DNA, *n* (%)	Any	58 (48.3)	44 (54.3)	14 (35.9)	0.079
	NRTI only	3 (2.5)	1 (1.2)	2 (5.1)	—
	NNRTI only	5 (4.2)	4 (5.0)	1 (2.6)	—
	PI only	1 (1.0)	1 (1.2)	0 (0)	—
	NRTI + NNRTI	47 (39.2)	36 (44.4)	11 (28.2)	0.111
	NRTI + PI	1 (1.0)	1 (1.2)	0 (0)	—
	NRTI + NNRTI + PI	1 (1.0)	1 (1.2)	0 (0)	—
	None	32 (26.7)	16 (19.8)	16 (41.0)	0.017
	Not available	30 (25.0)	21 (25.9)	9 (23.1)	0.824
HIV-1 subtype, *n* (%)	CRF02_AG	53 (44.1)	35 (43.2)	18 (46.1)	0.845
	A1	13 (10.8)	10 (12.3)	3 (7.7)	0.544
	G	7 (5.8)	5 (6.2)	2 (5.1)	—
	Others^b^	17 (14.2)	10 (12.3)	7 (17.9)	0.415
	Not available	30 (25.0)	21 (25.9)	9 (23.1)	0.824

GFR, glomerular filtration rate; TDF, tenofovir disoproxil fumarate; ZDV, zidovudine; d4T, stavudine; PI/r, ritonavir-boosted PI; 3TC, lamivudine; LPV/r, ritonavir-boosted lopinavir; ABC, abacavir; ddI, didanosine; ATV/r, ritonavir-boosted atazanavir; IDV/r, ritonavir-boosted indinavir.

^a^Participants had experienced nevirapine (70/120; 58.3%) and/or efavirenz (65/120; 54.2%), without a significant difference between arms; PI/r experienced included LPV/r (118/120; 98.3%), IDV/r (13/120; 10.8%) and ATV/r (9/120; 7.5%), without significant differences between arms.

^b^Comprising CRF11_cpx (*n* = 3); B, F1 and CRF37_cpx (*n* = 2 of each); D, H, F2, CRF01_AE, CRF09, CRF06_cpx and CRF18_cpx (*n* = 1 of each) and unassigned subtype (*n* = 1).

### Resistance patterns in cellular HIV-1 DNA at study entry

HIV-1 DNA sequences were obtained from 90/120 (75%) participants (Table 1). Six participants did not have a PBMC sample and 24 did not yield a sequence in ≥2 attempts (until sample exhaustion). Overall 58/90 (64.4%) sequences showed ≥1 RAM, most commonly affecting the NRTIs (52/90; 57.8%) and the NNRTIs (53/90; 58.9%); 47/90 (52.2%) had RAMs for both classes (Table 1). Protease RAMs were uncommon; three participants showed the nelfinavir RAM D30N either alone or with RAMs to other classes; they had received ritonavir-boosted lopinavir for 1–3 years and had no prior nelfinavir exposure. Overall, 11/90 (12.2%) HIV-1 DNA sequences showed in-frame stop codons within RT, including 9 sequences containing RAMs; hypermutation was also common in both RT and Protease sequences containing RAMs (Table 2).

**Table 2.** Major RAMs in cellular HIV-1 DNA at study entry (*n* = 90) and in plasma HIV-1 RNA at virological rebound (*n* = 23)^a^

RAMs		HIV-1 DNA (*n* = 90)		HIV-1 RNA (*n* = 23)		
		Total *N* (%)	Sequence^b^	1%–14%	≥15%	Total *N* (%)
NRTIs	Any	52 (57.8)		4 (17.4)	6 (26.1)	9 (39.1)
	M41L	12 (13.3)	3 HM	1 (4.3)	3 (13.0)	4 (17.4)
	D67G	1 (1.6)		—	1 (4.3)	1 (4.3)
	D67N	9 (10)	2 SC, 1 HM	1 (4.3)	—	1 (4.3)
	K70R	14 (15.6)	5 SC	—	1 (4.3)	1 (4.3)
	L210W	7 (7.8)	3 HM	—	2 (8.7)	2 (8.7)
	T215F	10 (11.1)	3 SC, 2 HM	—	2 (8.7)	2 (8.7)
	T215Y	9 (10.0)	1 HM	—	2 (8.7)	2 (8.7)
	T215rev^c^	3 (3.3)	1 SC	1 (4.3)	—	1 (4.3)
	T219Q T219E T219N T219R	8 (8.9)	2 SC, 1 HM	—	—	—
	K65R	2 (2.2)	1 SC	—	—	—
	L74V	3 (3.3)	1 HM	—	—	—
	L74I	2 (2.2)		—	2 (8.7)	2 (8.7)
	M184V	41 (45.6)	3 SC, 3 HM	1 (4.3)	6 (26.1)	7 (30.4)
	M184I M184I/V	5 (5.6)	2 SC, 3 HM	—	—	—
	Q151M/L	3 (3.3)	1 SC	—	—	—
	T69ins	2 (2.2)	1 HM	—	—	—
	T69D/N	2 (2.2)		—	—	—
	T69N	—		2 (8.7)	—	2 (8.7)
NNRTIs	Any	53 (58.9)		3 (13.0)	7 (30.4)	10 (43.5)
	A98G	11 (12.2)	2 SC, 1 HM	—	2 (8.7)	2 (8.7)
	L100I	1 (1.1)	1 SC	—	—	—
	K101E	6 (6.7)	1 HM	1 (4.3)	2 (8.7)	2 (8.7)
	K101H K101P/T	2 (2.2)	1 HM	—	—	—
	K103N	26 (28.9)	3 SC, 1 HM	1 (4.3)	3 (13.0)	4 (17.4)
	V106A	4 (4.4)	1 SC, 2 HM	—	—	—
	V106M	—		—	1 (4.3)	1 (4.3)
	V108I	11 (12.2)	2 SC, 1 HM	—	1 (4.3)	1 (4.3)
	E138G E138K	4 (4.4)	2 SC, 1 HM	—	—	—
	Y181C	15 (16.7)	2 SC, 2 HM	—	1 (4.3)	1 (4.3)
	Y188L Y188C Y188F/H/L	4 (4.4)	1 HM	—	—	—
	G190A	11 (12.2)	3 SC, 3 HM	1 (4.3)	1 (4.3)	2 (8.7)
	H221Y	5 (5.5)	2 SC	—	1 (4.3)	1 (4.3)
	P225H	2 (2.2)		—	—	—
	F227L	4 (4.4)	1 SC	—	—	—
	M230I	5 (5.5)	3 SC, 1 HM	1 (4.3)	—	1 (4.3)
	M230L	1 (1.1)		—	1 (4.3)	1 (4.3)
	K238T	5 (5.5)	1 SC, 1 HM	—	—	—
	Y318F	2 (2.2)		—	—	—
PIs	D30N^d^	3 (3.3)	2 HM	2 (8.7)	—	2 (8.7)

SC, stop codon; HM, hypermutation; T215rev, T215 revertant.

^a^HIV-1 DNA sequences were obtained from PBMC by Sanger sequencing; HIV-1 RNA sequences were obtained from plasma by UDS (Illumina MiSeq) and are reported according to the frequency thresholds of 1%–14% and ≥15%.

^b^RT sequences with in-frame stop codons (positions 24, 42, 48, 71, 88, 120, 153, 212, 219, 229, 239) and RT and protease sequences with hypermutation, with the number of sequences affected, are indicated.

^c^Comprising T215S, T215C, T215I and T215V.

^d^D30N was the only RAM detected in Protease.

### Virological outcomes

At Week 24, proportions with HIV-1 RNA < 400 copies/mL (primary endpoint) were 72/81 (88.9%) in the ritonavir-boosted darunavir arm and 37/39 (94.9%) in the SOC arm (*P* = 0.50). Proportions with HIV RNA < 60 copies/mL through to Week 24 were 62/81 (76.5%) and 36/39 (92.3%), respectively (*P* = 0.04). In the ritonavir-boosted darunavir arm, 24/81 (29.6%) participants experienced virological rebound (confirmed or last available HIV-1 RNA ≥ 60 copies/mL) through to Week 48, including 16/81 (19.8%) who experienced virological failure (confirmed or last available HIV-1 RNA ≥400 copies/mL). In the SOC arm, 3/39 (7.7%) participants experienced virological rebound through to Week 24, including 1/39 (2.6%) participant with virological failure.

### Resistance patterns in plasma HIV-1 RNA at virological rebound

Sequences were obtained by UDS in 23 participants who experienced virological rebound (ritonavir-boosted darunavir arm *n* = 21; SOC arm *n* = 2). HIV-1 RNA levels at the time of testing were median 3.0 log_10_ copies/mL (range 2.0–4.1). NRTI and NNRTI RAMs were found in 9/23 (39.1%) and 10/23 (43.5%) samples, respectively and 8/23 (34.7%) samples had RAMs for both classes; most RAMs occurred at frequency ≥15% (Table 2). No participant had darunavir RAMs at either high or low frequency. Two participants, both in the ritonavir-boosted darunavir arm, showed the nelfinavir RAM D30N at low frequency (3%–4%); before starting ritonavir-boosted darunavir, they had received ritonavir-boosted lopinavir for >4 years and had no prior nelfinavir exposure. No stop codons or hypermutation were found in plasma sequences.

### Comparison of resistance patterns in HIV-1 DNA and HIV-1 RNA

**Figure 1.**
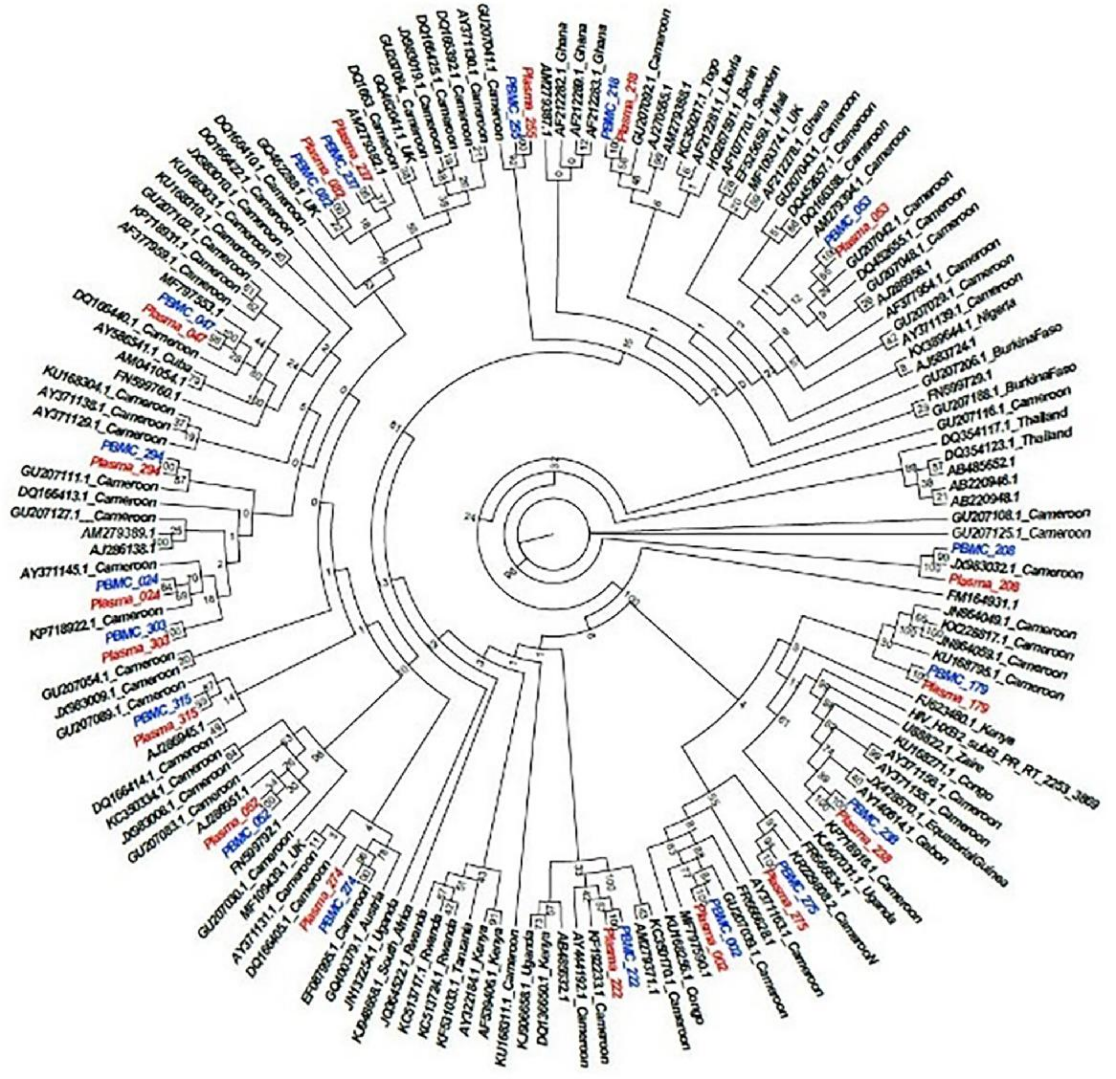
Maximum-likelihood phylogenetic tree of paired *pol* sequences of HIV-1 DNA obtained from PBMCs at study entry (blue) and HIV-1 RNA obtained from plasma at virological rebound (red) (*n* = 18 pairs). Control sequences were obtained from Genbank (duplicate sequences excluded). The tree was inferred using 1000 bootstrap replicates. This figure appears in colour in the online version of *JAC* and in black and white in the print version of *JAC*.

There were 18 participants with paired cellular HIV-1 DNA and plasma HIV-1 RNA sequences (Table 3). Phylogenetic analyses confirmed clustering of the *pol* sequences (Figure 1). With 6/18 (33.3%) sample pairs, the resistance patterns were fully consistent, comprising 5 with no RAMs in either sample and 1 with the NNRTI RAM K103N in both samples (Table 3). A further 6/18 (33.3%) sample pairs showed partial consistency, comprising 5 with fewer RAMs in HIV-1 DNA and 1 with fewer RAMs in HIV-1 RNA. With 5/18 (27.8%) sample pairs, RAMs were detected only in HIV-1 DNA; 3 HIV-1 DNA sequences showed in-frame stop codons and 1 with the NNRTI RAM M230I also showed hypermutation. One sample pair showed M230I in HIV-1 RNA only (frequency 1%). There was no consistency in the detection of the Protease RAM D30N when comparing sample pairs, and no linkage was detected between *pol* sequences from the five individuals with D30N (Figure S1).

**Table 3.** Paired cellular HIV-1 DNA sequences obtained at study entry and plasma HIV-1 RNA sequences obtained at virological rebound (*n* = 18 participants)^a^

	Study entry								Arm	Virological rebound				
ID	Years of prior exposure^b^					RAMs in HIV-1 DNA				Study week	HIV-1 RNA copies/mL	RAMs in HIV-1 RNA^c^		
	ART	TDF	TA	NNRTI	PI	NRTI	NNRTI	PI				NRTI	NNRTI	PI
024	10.9	4.9	0.0	5.8	10.6	None	None	None	DRV/r	48	410^f^	None	None	None
047	7.6	2.8	4.7	5.8	1.8	None	None	None	DRV/r	48	595^f^	None	None	None
053	1.9	1.7	0.7	0.2	1.9	None	None	None	DRV/r	12	5454^g^	None	None	None^j^
179	3.7	1.0	2.7	2.7	1.0	None	None	None	DRV/r	48	1506^f^	None	None	None
274	8.5	6.4	2.0	2.1	6.4	None	None	None	DRV/r	24	489^g^	None	None	None
238	7.4	5.1	2.3	1.7	5.7	None	None	None	DRV/r	48	1098^h^	None	M230I_(1)_	None
294	1.7	1.7	0.6	0.7	1.0	K70R	None	None	DRV/r	36	621^g^	None	None	None
275	5.6	2.7	2.8	4.3	1.3	None	K103N	None	DRV/r	24	13 381^g^	None	None	None^j^
082	7.0	1.7	5.8	5.7	1.2	None	K103N	None	DRV/r	24	13 024^g^	None	K103N_(1)_	None^j^
222	7.4	3.5	3.9	4.0	3.4	None	M230I^d,e^	None	DRV/r	12	823^i^	None	None	None
002	6.3	1.3	5.0	5.0	1.3	M184V T215F	K101E G190A	None	DRV/r	48	2707^f^	M41L_(2)_ M184V_(99)_ T215F_(67)_	K101E_(98)_ G190A_(99)_	None
208	9.0	4.4	4.5	4.6	4.4	M184V T215Y	A98G, Y181C	None	DRV/r	48	201^f^	M184V_(67)_ M41L_(60)_ T215Y_(24)_	A98G_(33)_ Y181C_(73)_ M230I_(9)_	D30N_(4)_
315	13.7	5.5	8.1	3.8	9.9	M41L M184V	K101E K103N	None	DRV/r	36	1608^h^	M41L_(99)_ T215Y_(95)_ T215C_(4)_	None	None
218	9.4	4.6	4.8	4.8	4.6	M41L L74I M184V L210W T215Y	A98G K103N P225H	None	DRV/r	24	105^i^	M184V_(53)_ T215F_(38)_	A98G_(21)_ K103N_(32)_ Y232H_(17)_	D30N_(3)_
303	7.7	1.7	4.0	4.0	3.7	M41L D67N K70R L74I M184V T215F K219R^d^	L100I K103N	None	DRV/r	12	1758^g^	None	None	None^j^
052	9.1	4.0	5.1	5.1	4.0	K65R K70R V75I F77L F116Y Q151LM M184I^d^	V108I E138G Y181C G190A H221Y	None	DRV/r	48	3715^f^	None	None	None^j^
255	1.8	1.7	0.1	0.2	1.75	M184V	None	None	SOC	24	173^f^	M41L_(99)_ L74I_(99)_ M184V_(99)_ L210W_(97)_	V108I_(99)_ H221Y_(95)_ M230L_(99)_	None
237	7.9	0.0	2.8	5.1	2.8	M184V	K103N	None	SOC	24	706^f^	M41L_(99)_ M184V_(99)_ L210W_(99)_	K103N_(99)_	None

TDF, tenofovir disoproxil fumarate; TA, thymidine analogues (zidovudine or stavudine).

^a^HIV-1 DNA sequences were obtained from PBMCs by Sanger sequencing; HIV-1 RNA sequences were obtained from plasma by UDS (Illumina MiSeq).

^b^Total exposure at study entry.

^c^The frequency of the mutant is indicated in parentheses.

^d^HIV-1 DNA sequences with in-frame stop codons.

^e^HIV-1 DNA sequences showing hypermutation.

^f^Last available HIV-1 RNA measurement.

^g^First HIV-1 RNA measurement of confirmed virological failure.

^h^Second HIV-1 RNA measurement of confirmed virological failure.

^i^First HIV-1 RNA measurement of confirmed virological rebound.

^j^Sample also underwent *gag* sequencing.

### Gag mutations

**Figure 2.**
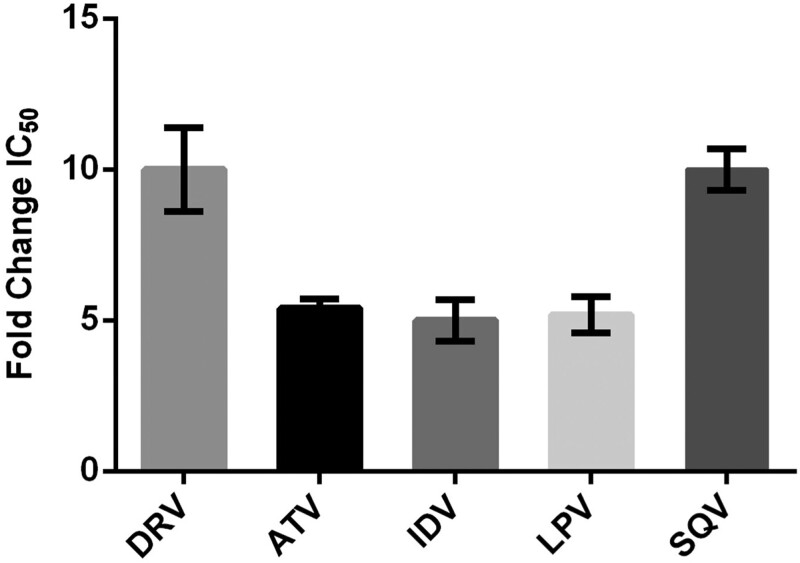
Phenotypic resistance profile of the *gag* p2/p7 cleavage site mutation T375A, as determined by site-directed mutagenesis. The mean FC in IC_50_ from three independent experiments (with standard deviation) is shown. ATV, atazanavir; DRV, darunavir; IDV, indinavir; LPV, lopinavir; SQV, saquinavir.

Among participants who experienced virological rebound on ritonavir-boosted darunavir, eight underwent *gag* sequencing, including four who were tested longitudinally (Table 4). None of the sequences contained RAMs in Protease. Screening for Gag mutations significantly associated with PI exposure revealed four sequences with five cleavage site mutations. One participant with longitudinal samples showed emergence of K436R in p7/p1 between Week 24 and Week 36, when K436R replaced T375N in p2/p7. Two sequences showed T375A in p2/p7, in one case occurring with K436R. The phenotypic effects of T375A were analysed by site-directed mutagenesis. The mutation reduced PI susceptibility by approximately 5-FC for atazanavir, indinavir and lopinavir and 10-FC for darunavir and saquinavir (Figure 2). The mutation also increased replicative capacity to 161% (±5.0) relative to WT control. Furthermore, all sequences showed mutations within other Gag regions. Among participants with longitudinal results, emergent mutations included D121G in p17, T190I in p24 and P478Q in p6.

**Table 4.** Gag mutations in plasma HIV-1 RNA of participants who experienced virological rebound during darunavir/ritonavir monotherapy^a^

ID^b^	Years of exposure^c^		Study week	HIV-1 RNA copies/mL	HIV-1 subtype	p17				*p17* *p24*	p24			*p24* *p2*	p2	*p2* *p7*	p7	*p7* *p1*	p1	*p1* *p6*	p6			
	ART	PI																						
052	9.1	4.0	48	3715	CRF_02AG				N126S		T186M	T190I	S310T			*T375A*		*K436R*				T469I		
053	1.9	1.9	12	5454	CRF_02AG			D121A			T186M				T371Q						F463L			
053			24	15 756				D121A			T186M	T190I									F463L			
082	7.0	1.2	24	13 024	CRF_02AG			D121A	N126S		T186M	T190I	S310T		T371Q						F463L			T491I
082			36	1137				D121A	N126S		T186M	T190I	S310T		T371Q						F463L			T491I
243	3.8	1.2	24	23 933	CRF_02AG		K113Q		N126S		T186M	T190I	S310T		T371Q							T469I		
243			36	4376			K113Q	D121G	N126S		T186M	T190I	S310T		T371Q							T469I	P478Q	
274	8.5	6.4	36	4005	CRF_02AG		K113Q				T186M	T190I	S310T		T371Q	*T375A*						T469I		
275	5.6	1.3	24	13 381	A1	I94V	K113Q		N126S		T186M	T190I	S310T			*T375N*						T469I	P478Q	
275			36	30 762		I94V	K113Q		N126S		T186M	T190I	S310T					*K436R*				T469I	P478Q	
303	7.7	3.7	12	1758	CRF_02AG				N126S		T186M		S310T									T469I		
315	13.7	9.9	12	1156	CRF_02AG			D121G			T186M	T190I	S310T		T371Q									

^a^Gag mutations (relative to HIV-1 HXB2) significantly associated with PI exposure are shown, as pre-defined by comparing full-length Gag-Protease sequences from 200 PI-naive and 191 PI-experienced individuals; Gag cleavage sites *are in italics*; there were no RAMs in Protease.

^b^In 4 participants, two longitudinal samples were tested.

^c^Total exposure at study entry.

## Discussion

We studied a population in Cameroon that following failure of first-line NNRTI-based ART started two NRTIs plus a ritonavir-boosted PI (typically ritonavir-boosted lopinavir) in the absence of virological monitoring. After confirmation of virological suppression, participants were assigned to either maintenance monotherapy with ritonavir-boosted darunavir or tenofovir disoproxil fumarate/lamivudine plus ritonavir-boosted lopinavir. Although interpretation is limited by the small number of participants, the findings align with published data from a similar population,^6^ indicating a heightened risk of viraemia among individuals on ritonavir-boosted darunavir monotherapy. Over 48 weeks, we observed several instances of virological rebound (confirmed or last available HIV-1 RNA ≥ 60 copies/mL) and virological failure (HIV-1 RNA ≥ 400 copies/mL) in this group, with nearly 30% of participants experiencing rebound viraemia. Of note, the study adopted an HIV-1 RNA threshold of 400 copies/mL to define virological failure in line with similarly designed clinical trials conducted in the region.^6^ At virological rebound, the resistance patterns in plasma HIV-1 RNA were partially reflective of those detected in cellular HIV-1 DNA at study entry. UDS confirmed the absence of treatment-emergent darunavir RAMs in Protease. However, we detected mutations in Gag and demonstrated an effect on darunavir susceptibility in the absence of RAMs in Protease.

In cell culture, two pathways of darunavir resistance have been characterized anchored around the Protease RAMs I50V or I84V.^20^ Darunavir RAMs have also been shown to emerge in PI-experienced people with pre-existing Protease RAMs but have rarely occurred when starting ritonavir-boosted darunavir *de novo*; the high resistance barrier is thought to reflect tight binding of darunavir to the Protease enzyme and high plasma concentrations achieved through pharmacological boosting.^20^ Monotherapy studies have also reported a negligible risk of darunavir RAMs.^5^ Most studies employed Sanger sequencing for detecting resistance, but two studies applied more sensitive techniques. One study using single-genome sequencing in five participants with HIV-1 RNA > 400 copies/mL detected darunavir RAMs (V32I, I47V, I50V) in one participant lacking the mutations by Sanger sequencing.^21^ A second study using UDS in 14 participants with HIV-1 RNA > 1000 copies/mL found I54T in the Protease of a participant lacking Protease RAMs by Sanger sequencing;^22^ however, I54T is not considered a darunavir RAM.^11^ We complement these data by reporting UDS results from 21 participants and by extending the analysis to all cases of confirmed or last available plasma HIV-1 RNA ≥ 60 copies/mL, including 12 with viral load< 1000 copies/mL. No darunavir RAMs were detected in the 21 participants.

Two participants on ritonavir-boosted darunavir showed the Protease RAM D30N in rebound HIV-1 RNA but not in HIV-1 DNA at study entry. The significance is doubtful. D30N is a non-polymorphic substrate-cleft mutation that is selected by and causes high-level resistance to nelfinavir.^23^ The aspartate-to-asparagine substitution alters the hydrogen bond interaction with the aniline NH_2_ group of darunavir, causing some loss of binding affinity.^24^ However, the mutation occurred only at low frequency (<5%) in rebound HIV-1 RNA. Furthermore, there is no evidence of an association with darunavir resistance.^11^ At study entry, D30N was also detected in the HIV-1 DNA of three ritonavir-boosted lopinavir-experienced participants, but there is similarly no evidence that ritonavir-boosted lopinavir selects for D30N.^23^ PCR-induced error or APOBEC3G-mediated hypermutation, an innate defence mechanism that aims to impair virus functionality, may also explain the unexpected detection of D30N.^25^ Substitutions that have been related to hypermutation include D30N and M46I in Protease and E138K, M184I, G190E and M230I in RT.^25–27^ We found hypermutation in several HIV-1 DNA sequences showing these mutations.

Mutations in Gag, the natural substrate of the Protease enzyme, can improve Gag–Protease binding and Gag processivity and modulate PI susceptibility and viral fitness in the absence of or alongside Protease RAMs.^28–34^ Mutations associated with PI exposure typically involve Gag cleavage sites, although mutations in other regions have also been implicated.^18,33,34^ We had sufficient sample for full-length *gag* and *protease* sequencing in a subset of participants experiencing virological rebound on ritonavir-boosted darunavir. All had Gag mutations previously associated with PI exposure, including mutations at the cleavage sites p2/p7 and p7/p1 and within p17, p24, p2 and p6. In one case, we were able to demonstrate emergence of K436R in p7/p1 while HIV-1 RNA levels ranged between 13 381 and 30 762 copies/mL on ritonavir-boosted darunavir. K436R was associated with PI exposure and resistance in previous studies and was shown to occur during ritonavir-boosted darunavir monotherapy.^28,33^ We also detected T375A in p2/p7 in two participants. T375A was previously detected in an individual receiving ritonavir-boosted darunavir monotherapy in combination with S451N in p1/p6 and several additional Gag mutations.^33^ Given the limited data available, we produced a site-directed mutant with T375A and observed a 10-FC reduction in darunavir susceptibility, alongside increased viral replication capacity. Outside of cleavage sites, we saw emergence of D121G in p17, T190I in p24 and P478Q in p6. T190I is an HLA-B*81-associated mutation with a compensatory role in viral fitness.^35^ Regrettably, we were unable to expand the *gag* analyses due limited sample volumes. Of note, we focused the analysis of Gag sequences on mutations previously found to be associated with PI exposure, using strict criteria to define the association. This approach may not account for all possible Gag mutations that may contribute to darunavir resistance. In fact, significant mutations may not necessarily be confined to consistent changes at a few sites.^36^ Importantly, all participants with Gag mutations regained virological suppression after receiving adherence support and returning to ritonavir-boosted PI-based triple ART.

There is growing interest in the potential clinical utility of sequencing cellular HIV-1 DNA to guide treatment decisions during virological suppression.^2^ As we previously reported on a smaller subset,^4^ the resistance patterns detected in HIV-1 DNA at study entry were consistent with the previous failure of NNRTI-based ART. The resistance patterns detected in rebound HIV-1 RNA on ritonavir-boosted darunavir were generally in agreement with those found at study entry, but discrepancies were common. This is not unexpected. Several HIV-1 DNA sequences containing RAMs were defective and could not be expected to sustain virus production.^37^ A further consideration is that in the setting of virological suppression the HIV-1 DNA input into the sequencing test is small and detection of RAMs can become stochastic. Due to limited infrastructure, we did not perform UDS on PBMCs, which may have increased the detection of RAMs in HIV-1 DNA,^38^ although even UDS cannot be expected to overcome issues of input size and provide a full representation of the resistance archive.^2^ Furthermore, the clinical significance of detecting low-frequency RAMs, particularly in the context of regimens with a high barrier to resistance, remains doubtful.

In summary, the resistance patterns of cellular HIV-1 DNA sequenced during virological suppression, whilst largely reflective of treatment history, are only partially consistent with the resistance patterns of rebound viraemia. Both biological and technical factors may account for the discrepancies and should be taken into consideration when applying the test clinically. We confirm the high resistance barrier of ritonavir-boosted darunavir but demonstrate that Gag substitutions, including T375A in in p2/p7, provide an alternative pathway of darunavir resistance.

## Supplementary Material

dkad386_Supplementary_Data

## Supplementary data

Figure S1 and Table S1 are available as Supplementary data at *JAC* Online.
