# Supplementary material for: A detailed characterization of drug resistance during darunavir/ritonavir monotherapy highlights a high barrier to the emergence of resistance mutations in protease but identifies alternative pathways of resistance
Source: J Antimicrob Chemother. 2023 Dec 28;79(2):339–48. doi: 10.1093/jac/dkad386 (PMC10832591; doi:10.1093/jac/dkad386)
Supplement: dkad386_Supplementary_Data [file dkad386_supplementary_data.docx]

**Supplementary Table 1.** Gag sequencing conditions

| **RT-PCR** |  |  |  |
| --- | --- | --- | --- |
| **Reagent** |  | **Amount per sample (**µ**l)** | |
| 2x reaction mix |  | 25 |  |
| GAGBOUT (10µM) |  | 1 |  |
| GAGFOUT (10µM) |  | 1 |  |
| Superscript III RT/Platinum *taq* high fidelity enzyme mix | | 1 |  |
| Water |  | 7 |  |
| **Conditions** | **Temperature (^o^C)** | **Time** | **Cycles** |
| cDNA synthesis | 55 | 30 minutes | 1 |
| Denaturation | 94 | 2 minutes | 1 |
| Denature | 94 | 15 secs | 40 |
| Anneal | 60 | 30 secs |  |
| Extend | 68 | 2 minutes |  |
| Final extension | 68 | 5 minutes | 1 |
| Hold | 4 | (<24 hours) | 1 |
| **Nested PCR** |  |  |  |
| **Reagent** |  | **Amount per sample (μl)** | |
| Primer GAGFIN |  | 1 |  |
| Primer GAGBIN |  | 1 |  |
| Platinum PCR SuperMix high fidelity | | 45 |  |
| **Conditions** | **Temperature (^o^C)** | **Time** | **Cycles** |
| Initial denaturation | 94 | 2 minutes | 1 |
| Denature | 94 | 30 secs | 40 |
| Anneal | 52 | 30 secs |  |
| Extend | 68 | 2 minutes |  |
| Hold | 4 | (<24 hours) | 1 |
| **Primer Name** | **Sequence** | **HXB2 Position** | **Primer description** |
| GAGFOUT | GTTGTGTGACTCTGGTAACTAGAGATCCCTCAGA | 570-603 | Forward outer |
| GAGBOUT | TCCTAATTGAACYTCCCARAAGTCYTGAGTTC | 2797-2828 | Reverse outer |
| GAGFIN | TCTCTAGCAGTGGCGCCCGAACAG | 626-649 | Forward inner |
| GAGBIN | GGCCATTGTTTAACCTTTGGDCCATCC | 2597-2623 | Reverse inner |
| Alternate nested primers | | | |
| GAGFIN2 | AAATCTCTAGCAGTGGCGCCCGAACAG | 623-649 | Forward inner |
| GAGBIN2 | TGGMCCAAARGTTAAACARTGGC | 2600-2622 | Reverse inner |
| Sequencing primers | | | |
| G00 | GACTAGCGGAGGCTAGAAG | 764-782 | Forward |
| G50 | CACAGCAAGCAGCAGCTG | 1133-1150 | Reverse |
| G70 | ATGAGGAAGCTGCAGAATGGG | 1406-1426 | Forward |
| G01 | AGGGGTCGTTGCCAAAGA | 2281-2264 | Reverse |
| G05 | TGTTGGCTCTGGTCTGCTCT | 2157-2138 | Reverse |
| G35 | CATGCTGTCATCATTTCTTCTA | 1838-1817 | Reverse |
| G45 | TTGGACCAACAAGGTTTCTGTC | 1761-1740 | Reverse |
| G85 | TGC ACT ATA GGG TAA TTT TG | 1193-1173 | Reverse |

**Supplementary Figure 1.** Maximum-likelihood phylogenetic tree to evaluate linkage between sequences with the D30N mutation in protease (1000 bootstrap replicates). There were 5 participants that showed
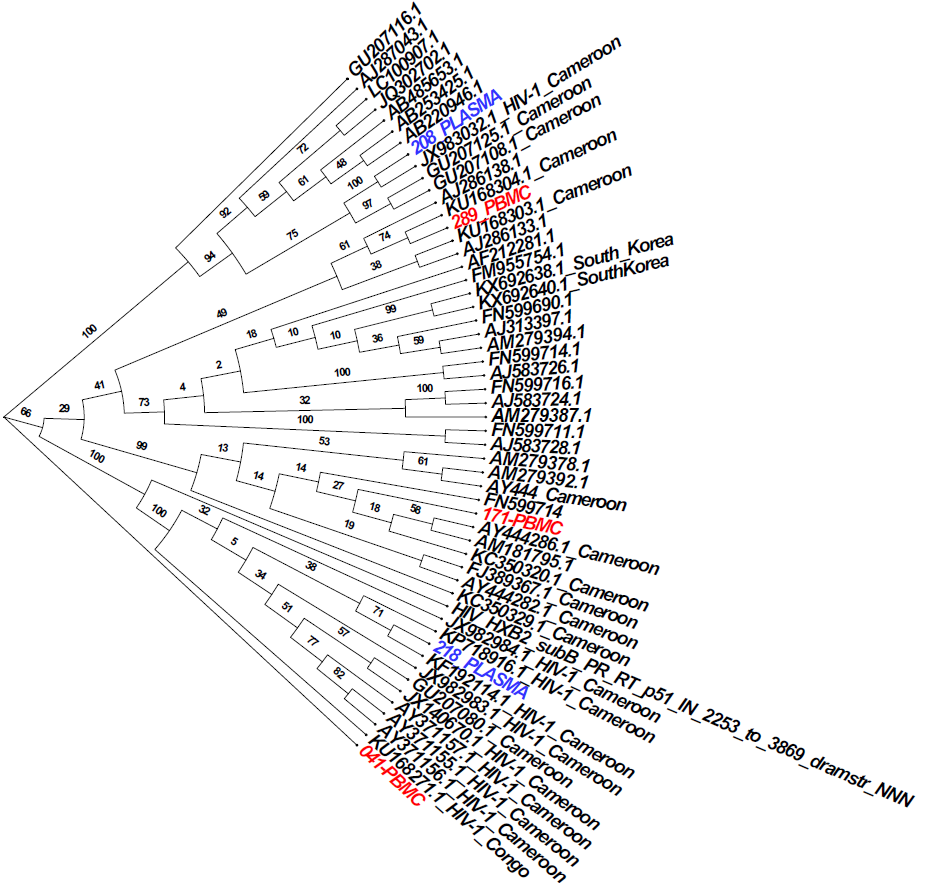
D30N in either cellular HIV-1 DNA (in red; n=3) or in plasma HIV-1 RNA (in blue; n=2)
